# High-performance achromatic flat lens by multiplexing meta-atoms on a stepwise phase dispersion compensation layer

**DOI:** 10.1038/s41377-024-01731-8

**Published:** 2025-03-05

**Authors:** Jingen Lin, Jinbei Chen, Jianchao Zhang, Haowen Liang, Juntao Li, Xue-Hua Wang

**Affiliations:** 1 State Key Laboratory of Optoelectronic Materials and Technologies, School of Physics, Sun Yat-Sen University, Guangzhou, China; 2 Hisense Laser Display Co.,Ltd, 399 Songling Road, Qingdao, Shandong China; 3 Quantum Science Center of Guangdong-Hong Kong-Macao Greater Bay Area (Guangdong), Shenzhen, China

**Keywords:** Metamaterials, Nanophotonics and plasmonics

## Abstract

Flat optics have attracted interest for decades due to their flexibility in manipulating optical wave properties, which allows the miniaturization of bulky optical assemblies into integrated planar components. Recent advances in achromatic flat lenses have shown promising applications in various fields. However, it is a significant challenge for achromatic flat lenses with a high numerical aperture to simultaneously achieve broad bandwidth and expand the aperture sizes. Here, we present the zone division multiplex of the meta-atoms on a stepwise phase dispersion compensation (SPDC) layer to address the above challenge. In principle, the aperture size can be freely enlarged by increasing the optical thickness difference between the central and marginal zones of the SPDC layer, without the limit of the achromatic bandwidth. The SPDC layer also serves as the substrate, making the device thinner. Two achromatic flat lenses of 500 nm thickness with a bandwidth of 650–1000 nm are experimentally achieved: one with a numerical aperture of 0.9 and a radius of 20.1 µm, and another with a numerical aperture of 0.7 and a radius of 30.0 µm. To the best of our knowledge, they are the broadband achromatic flat lenses with highest numerical apertures, the largest aperture sizes and thinnest thickness reported so far. Microscopic imaging with a 1.10 µm resolution has also been demonstrated by white light illumination, surpassing any previously reported resolution attained by achromatic metalenses and multi-level diffractive lenses. These unprecedented performances mark a substantial step toward practical applications of flat lenses.

## Abstract (web summary)

We demonstrate theoretically and experimentally that the stepwise phase dispersion compensation layer releases the numerical aperture, operation bandwidth, and aperture size from the tradeoffs of achromatic flat lens, allowing their flexible design.

## Graphical abstract

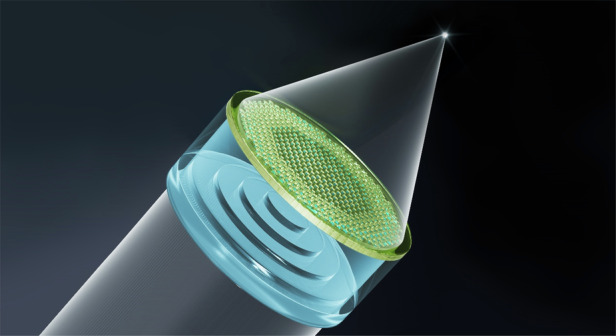

## Introduction

Miniaturized, ultracompact, and lightweight optical systems^1–6^ are increasingly desired in modern demands. One of the crucial elements in these systems are the optical lenses that focus light achromatically. Although achromatic lenses were developed centuries ago, they necessitate the assembly of multiple components to control light dispersion. It results in bulky and intricate systems that fail to meet the requirements of miniaturization, ultra-compactness and light weight. Therefore, single-lens systems that simultaneously deliver multiple high optical performances, such as wide achromatic bandwidth, high numerical aperture (NA), and a slim form-factor, emerge as promising candidates to satisfy contemporary demands. Thanks to the fast development of flat optics^7–9^ which are able to flexibly manipulate light in thin film layers, the focusing ones—the flat lenses, stand out as ideal candidates for such single-lens systems^10,11^. Unlike traditional optical lenses, flat lenses excel in delivering precise and efficient phase control^12–15^. This capability allows for the manipulation of transmitted or reflected light, enabling the achievement of specific functionalities, showing successes in various fields such as light-field imaging^16^, quantum optics^17–19^, biophysics^20^, extreme ultraviolet lithography^21^, wearable optics^22^, and machine vision^23^. As the incident light is chromatic, it also requires achromatic focusing by flat lenses to ensure clear imaging^24,25^. However, chromatic aberrations tend to be more pronounced in flat lenses compared to traditional optical lenses due to their diffractive nature.

Achromatic metalenses represent a category of flat lenses constructed from sub-wavelength meta-atoms^26–32^. These meta-atoms arrange within a single layer and display different phase responses across various wavelengths, enabling the correction of chromatic aberration through the adjustment of wavelength-dependent phase dispersion. By utilizing the same diffraction order, the metalens focuses all wavelengths at a single point, effectively countering the issue of chromatic aberration. It is important to note that achromatic metalenses have faced the constraint regarding their NA, operating bandwidth, and size due to the limited capabilities in the phase dispersion of meta-atoms^33–36^. Especially for a high NA, this leads to a trade-off between the aperture size and the bandwidth of the achromatic metalenses. Many previous studies have focused extensively on expanding the phase dispersion capabilities of the meta-atoms^27,28,37^ to overcome such limitation. However, this improvement is very limited, and inevitably complicates both the development of the meta-atom library and the fabrication process. An alternative type of achromatic flat lens is the achromatic multi-level diffractive lens (MDL)^38,39^. For instance, large-scale achromatic MDLs have been designed and demonstrated to operate over a wide wavelength range with lens sizes up to 1 centimeter, but very small NAs of 0.1^39^. The MDLs are capable of focusing different wavelengths at the same focal point by utilizing various diffraction orders instead of the same diffraction order. Therefore, they come with significant challenges when trying to achieve high NAs, including the high complexity of fabrication and a notable decrease in efficiency.

The aforementioned approaches eliminate chromatic aberration within a single layer, which requires the meta-atoms to exhibit very high phase dispersion, ultimately limiting optical performance. In contrast, leveraging the advantages of hybrid or multilayer optics is expected to effectively enhance imaging capabilities by combining multiple optical elements or layers^40–45^. Combining separated bulk refractive lenses and metasurfaces is possible to correct chromatic focusing errors by utilizing their complementary negative and positive dispersions. For example, in Ref. ^42^, researchers used bulk refractive optical elements for focusing and incorporated metasurfaces to tailor the group delay and broaden the achromatic bandwidth of the bulk lens, which comes at the expense of the flattening and miniaturization. Hybrid and multilayer lenses fabricated via laser writing were proposed as strategies to modulate phase dispersion and achieve wide bandwidth achromatic focusing^41,43,44^. However, the relatively low accuracy of laser writing limits the aperture sizes and performances of these lenses in the visible and near-infrared (NIR) ranges. Additionally, the doublet metalens^45^ with both high NA and large aperture size was presented only for three specific discrete wavelengths by increasing the vertical degrees of freedom of the meta-atoms to match the focusing phase profiles. This approach significantly differs from broadband achromatism, where continuous phase compensation across a wide spectrum is required. Therefore, although many significant advances in the flat lenses have been made, it is highly desirable to find a strategy for creating the high NA and broadband achromatic flat lens with scale-up size and high-resolution imaging in either the visible or NIR ranges.

**Fig. 1:**
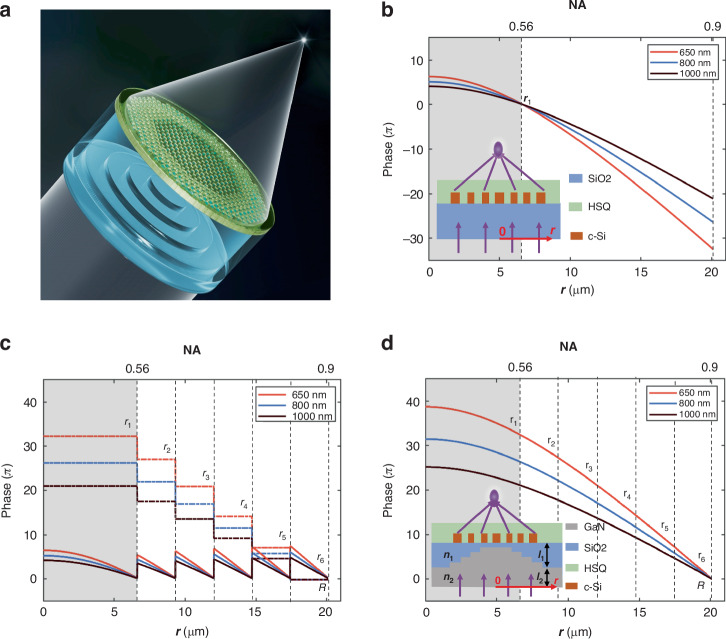
Design of achromatic flat lens with high NA. **a** Schematic of an achromatic flat lens. **b** Phase profiles of the conventional achromatic metalens. The focus length *f* and the maximum phase dispersion of the meta-atoms are set to be 9.7 μm and 2.20π, respectively. The achromatic operating is set within the wavelength range of 650 nm to 1000 nm. The gray region indicates the inherent size limitation of this achromatic flat lens if it is solely composed of conventional metalens. The corresponding maximum radius and NA of the conventional achromatic metalens are *r*_0_ = 6.6 μm and NA = 0.56, respectively. Inset is the schematic of the metalens. **c** The phase profiles of the meta-atoms *φ*_*meta*_ (*r*, *ω*) (solid lines), the relative phase Δ*ω*Δ*L*(*r*)/*c* corresponding to the SPDC layer (dashed lines) and (**d**) the relative phase *φ*_*lens*_ (*r,ω*) – *φ*_*lens*_ (*R,ω*) of the achromatic flat lens shown in (**a**). The parameters are identical to those in Fig. 1b. The corresponding maximum radius and NA of the flat lens are *r*_6_ = 20.1 μm and NA = 0.9, respectively. If using a conventional achromatic metalens to achieve the phase control of (**d**), then the required maximum phase dispersion of the meta-atoms would need to reach ΔΦ_*meta*_ = 13.60π. Inset is the schematic of the achromatic flat lens

In this work, we present the zone division multiplexing strategy of the meta-atoms integrated on a phase dispersion compensation layer partitioned by stepwise optical thickness to simultaneously reach broad achromatic bandwidth and expand the aperture size at a high NA, as shown in Fig. 1a. In this strategy, the stepwise phase dispersion compensation (SPDC) layer modulates the phase by the thickness difference between two materials, different from the previous methods of modulating phase. It can accumulate the extra phase at will by increasing the thickness difference of the SPDC layer, and is capable of compensating for the phase dispersion of the meta-atoms across any wide spectrum. Therefore, the radius of the flat lens can be on-demand enlarged, which facilitates the creation of a high-performance achromatic flat lens. Meanwhile, it also serves as an indispensable substrate without adding additional thickness.

Based upon this idea, we have experimentally fabricated an achromatic flat lens with the NA of 0.9, the radius of 20.1 μm, and very thin device thickness, which spans the entire first NIR window, covering wavelengths from 650 nm to 1000 nm by using a maximum meta-atoms phase dispersion of only 2.20π. For the conventional achromatic metalens, such high performance requires an unachievable phase dispersion of up to 13.60π, while the phase dispersion of 2.20π only creates the achromatic flat lens with the NA of 0.56 and the radius of 6.6 μm. Meanwhile, the NA of the fabricated flat lens is significantly higher than that of the achromatic MDLs. Furthermore, the broadband microscopic imaging with high resolution of 1.10 µm has been demonstrated by using the zone division multiplexing achromatic flat lens with the NA of 0.7 and a radius of 30.0 μm. In contrast, both the conventional achromatic metalenses and MDLs face challenges in achieving imaging resolutions finer than 2 μm. We report the broadband achromatic flat lenses with the recorded performances in the NA, size, thickness, and imaging resolution. Our approach introduces a new avenue for addressing the existing limitations and challenges associated with creating high-performance achromatic flat lenses.

## Results

### Principle

We investigate the flat lens constructed by the meta-atoms on a SPDC layer with varying optical thickness, which satisfy the achromatic conditions of the spherical aberration elimination^26,29,46^. Assuming the radius of the flat lens is *R*, we choose the phase of meta-atoms at *R* as zero within the achromatic range, then the phase profile of the achromatic flat lens *φ*_*lens*_ (*r,ω*) satisfies the following condition:

1 $$\begin{array}{c}{\varphi }_{lens}(r,\omega )={\varphi }_{meta}(r,\omega )+\frac{\omega }{c}[L(r)-L(R)]=\frac{\omega }{c}\left[\sqrt{{R}^{2}+{f}^{2}}-\sqrt{{r}^{2}+{f}^{2}}\right]\end{array}$$

where *φ*_*meta*_ (*r,ω*) is the phase profile contributed by the meta-atoms at the radial position *r*; *ω* is the frequency of the light; *L*(*r*) = $\bar{n}$(*r*) *l* is the optical thickness of the SPDC layer at *r*; $\bar{n}$(*r*) and *l* are the average refractive index at *r* and the thickness of this layer, respectively; *f* is the designed focal length. It is noted that the optical thickness of the SPDC layer imposes a very large initial phase into the actual phase of the flat lens. To eliminate this effect in the following analysis, we only consider the relative phase of the flat lens *φ*_*lens*_ (*r,ω*) – *φ*_*lens*_ (*R,ω*), i.e., the output phase of the flat lens induced by the meta-atoms and the optical thickness between its radius at *r* and *R*.

We denote the maximum phase dispersion that can be compensated by the meta-atom library as ΔΦ_*meta*_, and the value of the phase dispersion is positive^35^. The required phase dispersion of the meta-atoms from the phase profile suggested in Eq. (1) is then subject to the following restrictions:

2 $$\Delta {\varphi }_{lens}(r,\omega )=\Delta {\varphi }_{meta}(r,\omega )+\frac{\Delta \omega }{c}\Delta L(r)=\frac{\Delta \omega }{c}\left[\sqrt{{R}^{2}+{f}^{2}}-\sqrt{{r}^{2}+{f}^{2}}\right]$$

where Δ*φ*_*meta*_(*r*) is the required phase dispersion of the meta-atoms and its variation must be limited within $0\le \Delta {\varphi }_{meta}(r)\le \Delta {\varPhi }_{meta}$; Δ*ω* is the bandwidth of incident light, Δ*L*(*r*) = *L*(*r*) - *L*(*R*) is the optical thickness difference of the SPDC layer between its radius at *r* and *R*. Noticing from the right side of Eq. (1) that the maximum phase dispersion occurs at the center of the achromatic flat lens (*r* = 0), the maximum radius *R* of the achromatic flat lens can be obtained from Eq. (2):

3 $$\sqrt{{R}^{2}+{f}^{2}}={L}_{meta}+\Delta L(0)+f$$

where *L*_*meta*_ = *c*ΔΦ_*meta*_ / Δ*ω* is the meta-featured size, Δ*L*(*0*) the optical thickness difference between the center and edge of the flat lens. Evidently, Δ*L*(0) = 0 corresponding to the conventional metalens with a flat substrate, the radius of the flat lens is determined only by the meta-featured size *L*_*meta*_ and limited by the achromatic bandwidth.

Equation (3) provides new and effective strategies to expand the aperture size of the achromatic flat lens without compromising bandwidth Δ*ω* for a given ΔΦ_*meta*_ due to the introduction of the optical thickness Δ*L*(0). For the same focal length *f*, the radius of the achromatic flat lens could be in principle expanded at will by increasing Δ*L*(0). In Supplementary Discussion 1 and 4, we demonstrate that the radius of the achromatic flat lens can be freely enlarged by the identical step-thickness or identical step-width schemes. Of course, using meta-atoms capable of high phase dispersion leads to increase in the meta-featured size *L*_*meta*_.

### Design and experimental results

We begin with taking a conventional achromatic metalens as an example, where *L*(*r*) = *L*(*R*) is fixed as a constant^35^. In this case, as shown in the left side of Eq. (2), the required phase dispersion decreases with increasing *r* until reaching *R*, e.g. *R* = *r*_1_, where it achieves the maximum radius of the conventional metalens. Constrained by the practical fabrication conditions in our laboratory foundry, we use the meta-atom library with the maximum achievable phase dispersion of ΔΦ_*meta*_ = 2.20π. It is worth noting that meta-atoms with higher maximum phase dispersion are more beneficial for designing and fabricating the SPDC layer. We then use this library to design an achromatic flat lens with a focal length *f* = 9.7 μm, and a bandwidth from 650 nm to 1000 nm. The corresponding relative phase of the flat lens *φ*_*lens*_ (*r,ω*)–*φ*_*lens*_ (*R,ω*) is illustrated in Fig. 1b. As shown in Fig. 1b, when *r* > *r*_1_, Δ*φ*_*meta*_ < 0, which requires the meta-atoms to compensate the negative phase dispersion and is beyond the compensation capacity of the meta-atom library. If we expand the size of the achromatic metalens to a radius *R* of 20.1 μm, corresponding to an NA of 0.9, then the required maximum phase dispersion in the meta-atom library would need to reach ΔΦ_*meta*_ = 13.60π. For a conventional achromatic metalens in practice, such a design is extremely difficult to attain due to the excessively high phase dispersion exhibited by the meta-atoms, rendering experimental realization infeasible as well.

The key of expanding the lens radius is to eliminate the negative phase dispersion raised by the meta-atoms at specific locations. We then raise the idea of zone division multiplexing of the meta-atoms by dividing the flat lens into *N* zones according to the reduce of the optical thickness *L*(*r*) along the radial direction. Considering the discrete meta-atoms and the convenience of the actual fabrication of the SPDC layer, the discrete strategy is adopted in this work. In this way, the working radius of the designed flat lens *r*_N_ is equal to the radius *R* and it is significantly larger than the maximum radius *r*_1_ of a conventional metalens constructed by the same meta-atom library. Therefore, the above design can be achieved by an achromatic flat lens which multiplexes the same meta-atom library with a maximum phase dispersion of ΔΦ_*meta*_ = 2.20π. The SPDC layer is constructed according to the zone division of the phase profile of the meta-atoms, with the design details provided in Supplementary Discussion 1 of the Supplementary Information. Following the identical step-width scheme, each step size in the SPDC layer is precisely calculated, with a step size of 2.7 μm for the six steps. The achromatic flat lens is then designed by multiplexing the meta-atom library six times on the specific division zones on the SPDC layer. It is significant to note that more steps can be incorporated into this design to achieve a larger radius, as discussed in the previous section that our method allows for on-demand expansion of the aperture size. The thickness of each step is designed by the identical step-width scheme according to Eq. S16 (as detailed in Supplementary Discussion 1), resulting in a calculated total thickness of the SPDC layer of 11.76 μm. According to the design, the phase profile contributed by the meta-atoms (as shown the solid lines of Fig. 1c) is discontinuous; therefore, the relative phase Δ*ω*Δ*L*(*r*)/c imposed by the optical thickness difference of the SPDC layer should also be discontinuous (as illustrated by the dashed lines in Fig. 1c) so that the relative phase of the flat lens *φ*_*lens*_ (*r,ω*) – *φ*_*lens*_ (*R,ω*) is continuous. Figure 1d suggests that this achromatic flat lens successfully enhances the maximum NA from 0.56 to 0.9 and expands the radius from 6.6 μm to 20.1 μm by multiplexing the same meta-atoms library, surpassing the capabilities of a conventional achromatic metalens. This improvement is attained without compromising the achromatic wavelength range from 650 nm to 1000 nm.

**Fig. 2:**
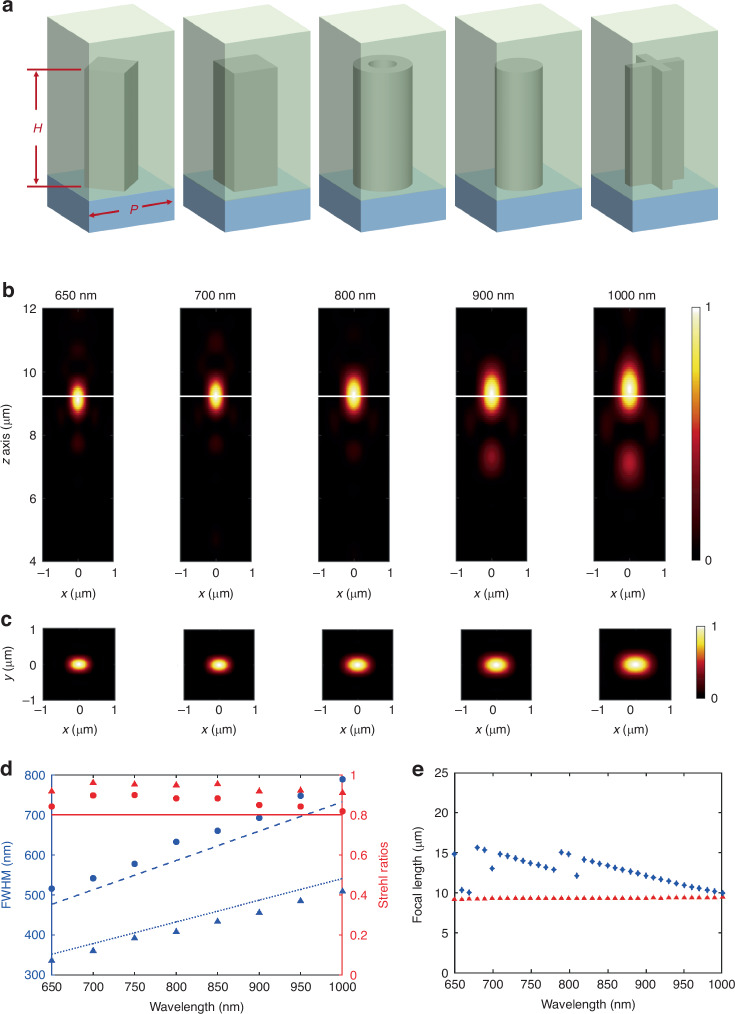
Simulation of achromatic flat lens with NA = 0.9 and a radius of 20.1 μm. **a** Illustration of five symmetric-shaped meta-atoms composed of crystalline silicon (c-Si) nanostructures, with the height *h* = 500 nm and the periodicity *p* = 300 nm. **b** The longitudinal PSFs of the achromatic flat lens at different incident wavelength. The white lines indicate the focal plane. **c** The corresponding transverse PSFs at the focal plane. **d** Calculated FWHM and SR at different incident wavelength for the achromatic flat lens at x direction (round dots) and y direction (triangle dots). The solid, dashed and dotted lines represent the SR above 0.8, theoretical FWHM calculated through VDIM in x direction and y direction, respectively. **e** The focal lengths as a function of wavelength for the achromatic flat lens (red dots) and a conventional achromatic metalens (blue dots)

Then the meta-atoms are simulated as in Fig. 2a. To assess the achromatic performance of the flat lens, finite difference time-domain (FDTD) simulations are performed, employing a horizontally linearly polarized (x-polarized) incident beam. The details of simulation are referred to the Simulation section in Methods. The numerical simulation results are depicted in Fig. 2b–e. Figure 2b, c display the point spread functions (PSFs) of the focal spot, covering a wavelength range from 650 nm to 1000 nm. It has been observed that the input linearly polarized beam is focused by the lens into an elliptical spot. This phenomenon is commonly associated with high NA lenses and originates from the vector characteristics of focused spot. In this case, we use Richards-Wolf vector diffraction integration method (VDIM) to calculate and characterize the diffraction limit focused spot of the ideal lens with high NA, rather than the Airy disk mode^47,48^. As depicted in Fig. 2d, the full width at half maximum (FWHM) and Strehl ratios (SR) of the achromatic flat lens meet the diffraction limit across the full spectrum of operational wavelengths. The red dots in Fig. 2e highlight the focal length and depth of focus (DOF) of the achromatic flat lens. For comparison, the blue dots in Fig. 2e depict the outcomes using a conventional achromatic metalens. The conventional achromatic metalens has a standard flat substrate, featuring an NA of 0.9, a radius of 20.1 μm, and an achromatic wavelength range from 650 nm to 1000 nm. It was designed following the previously reported approaches^26,29^, using five symmetrically shaped meta-atoms selected from the same meta-atom library as our achromatic flat lens. Evidently, our specially designed achromatic flat lens surpasses conventional metalens designs in correcting chromatic aberration. This clearly demonstrates that our flat lens achieves nearly ideal chromatic aberration correction and diffraction-limited focusing within its intended achromatic wavelength range, validating it highly suitable for high-performance imaging systems.

**Fig. 3:**
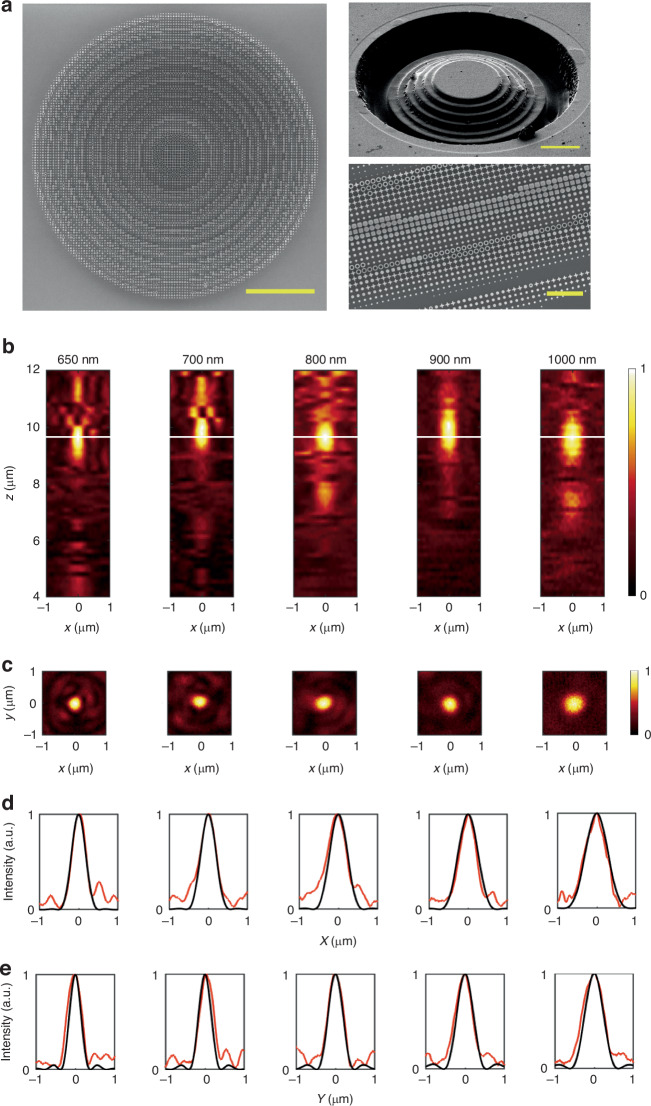
The experimentally fabricated achromatic flat lens with NA = 0.9 and a radius of 20.1 μm. **a** SEM image of the achromatic flat lens in top view (Scale bar, 10 μm). Right up: SEM image of the SPDC layer made of GaN (Scale bar, 10 μm). Right down: SEM image of a portion of the meta-atoms (Scale bar, 2 μm). The meta-atoms are patterned on the transferred c-Si layer. **b** Experimentally measured longitudinal PSFs at different incident wavelength by a x-polarized incident beam. The white lines indicate the focal plane. **c** The corresponding transverse PSFs at the focal plane. **d** The normalized intensity profiles at the focus plane in experimental (red lines) and in simulation through VDIM (black lines) at x direction. The FWHMs of the focal spot in experimental are respectively determined to be 401 nm, 458 nm, 495 nm, 524 nm, and 656 nm at wavelengths of 650 nm, 700 nm, 800 nm, 900 nm, and 1000 nm. **e** The normalized intensity profiles at the focus plane in experimental (red lines) and in simulation through VDIM (black lines) at y direction. The FWHMs of the focal spot in experimental are respectively determined to be 388 nm, 402 nm, 441 nm, 480 nm, 574 nm at wavelengths of 650 nm, 700 nm, 800 nm, 900 nm, and 1000 nm

Experimental fabrication and measurements were conducted to validate the performance of the achromatic flat lens, which has an NA of 0.9 and a radius of 20.1 μm. The fabrication process details are provided in the Fabrication section in Methods. The scanning electron microscope (SEM) image of the resulting structure is displayed in Fig. 3a. The measured PSFs across the wavelength range from 650 nm to 1000 nm by a x-polarized incident beam for the achromatic flat lens are depicted in Fig. 3b, c, with the details of the optical measurement setup available in the Optical characterization section in Methods. The measured focusing efficiencies are analyzed in Supplementary Discussion 3. Figure 3d, e further demonstrate the nearly diffraction-limited focusing within its intended achromatic wavelength range. Here the simulated results are calculated through VDIM by convolving the electric field of the PSF in the xy plane of the achromatic flat lens with the PSF of the microscope imaging system^45,47^.

The measured focusing efficiencies for high NA of 0.9 are about 10%, lower than the simulated focusing efficiencies from 22.4% to 29.1%. The difference between the theoretical and experimental results in our work is caused by the fabrication deviations of the staircase, as shown by SEM picture in Fig. 3a. Fortunately, the experimental efficiency of about 10% is adequate to get high-resolution imaging, as shown in the following imaging experiment. Also, it is noted that the efficiencies of the achromatic flat lenses dramatically decrease as the NA increases^46^, and their theoretical efficiencies at the NA of 0.9 range from 15% to 30%, which is lower compared to monochromatic flat lenses. From this perspective, the efficiency of our proposed flat lenses is consistent with the expectations. In the future, we will further develop the better fabricated technology to achieve the theoretical limit efficiency of the SPDC approach.

### Imaging performance

**Fig. 4:**
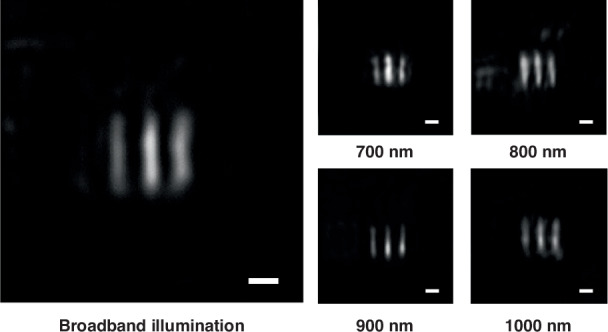
Imaging using an achromatic flat lens with NA = 0.7 and a radius of 30.0 μm. The microscope image of element 6, group 8 of the 1951 Unites States Air Force resolution target under broadband, 700 nm, 800 nm, 900 nm and 1000 nm illumination. Scale bars: 2 μm

A high NA single lens with a hyperbolic phase profile is susceptible to strong off-axis aberrations, which restricts the field of view (FOV). Consequently, achieving wide-field imaging is challenging with large NA single lenses, especially our achromatic flat lens with a diameter of only tens of micrometers. To evaluate the wide-field imaging capabilities, we conducted focusing and imaging tests on the achromatic flat lens with an NA of 0.7 and a radius of 30.0 μm because its field of view is sufficiently large for clear imaging, while the field of view of the metalens with an NA of 0.9 is too small to accommodate a resolution element within it. Detailed procedures are available in the Optical characterization section in Materials and methods. Figure S5. in the Supplementary Information shows the measured PSFs of this achromatic flat lens, and Fig. 4 displays the microscope imaging results of element 6 in group 8 of the United States Air Force resolution target under broadband illumination (650–1000 nm). The achromatic flat lens achieved a resolution of 1.10 μm. The distortion and blur of the elemental lines come from the monochromatic aberration at different wavelengths instead of chromatic aberration. During the image processing, adjustments were made by using multiscale retinex (MSR) to enhance image quality^49,50^ (refer to Fig. S6. in the Supplementary Information for the original images). It is also reported that using an asymptotic phase compensation strategy^27^ may effectively match the meta-atoms’ dispersion characteristics with the required complex phase dispersion profile, thereby further mitigating stray focus and enhancing the image quality.

## Discussion

Table 1 provides a comparison with previously reported experimental achromatic flat lenses in the visible and NIR wavebands for imaging purposes. Although we have fabricated the broadband achromatic flat lenses with the recorded performances in NA, aperture size, thickness, and imaging resolution, the performance can be further improved, especially in the aperture size. As analyzed in Supplementary Discussion 4, the radii of the flat lenses for any given focal length can be enlarged at will. Increasing the refractive index contrast of the SPDC layer, such as replacing GaN with crystalline silicon (c-Si), the step number of the SPDC layer can be dramatically decreased. Meanwhile, the thickness of the SPDC layer can be remarkably thinned. In this case, larger radius and higher NA are simultaneously obtained with much thinner thickness of each step, which is very advantageous for easier fabrication. Using meta-atoms with higher phase dispersion can further reduce the number of steps. These results demonstrate that our design can on-demand modulate the performance of the achromatic flat lens in a straightforward manner.

**Table 1:** Summary of reported experimental achromatic flat lenses in the visible and NIR wavebands for imaging

Reference	NA	Microscope Imaging Resolution (μm)^a^	Wavelength range (nm)	Radius(μm)	Maximum Phase dispersion^b^ ∆Φ	Total Device Thickness (nm)	Structure Type
This work	0.9 0.7	N/A 1.10	650–1000	20.1 30.0	2.20π	500 nm + substrate	Meta-atoms on a SPDC layer
Chen et al. ^26^	0.2 0.02	N/A 14.00	470–670	12.86 110	1.65π 1.40π	600 nm + substrate	Metalens
Hu et al. ^27^	0.164	8.77	420–1000	25	5.70π	1000 nm + substrate	Metalens
Wang et al. ^29^	0.106	3.10	400–660	25	2.62π	800 nm + substrate	Metalens
Wang et al. ^30^	0.24	2.19	650–1000	15	1.97π	1500 nm + substrate	Metalens
Shrestha et al. ^35^	0.88	N/A	1200–1400	50	7.10π	1400 nm + substrate	Metalens
Meem et al. ^38^	0.3	2.19	450–1000	1572.5	N/A	2600 nm + substrate	MDL
Xiao et al. ^39^	0.1	12.40	400–1100	5120	N/A	15000 nm + substrate	MDL
Richards et al. ^41^	0.3	3.91	400–700	45	N/A	14700 nm + substrate	Hybird microlens
Pan et al. ^43^	0.7 0.5	N/A 6.20	400–800	10 10	N/A	7800 nm + substrate	Multilayer diffractive lens

^a^ Data from the highest resolution achieved in imaging the USAF1951 resolution target as reported in reference

^b^ The maximum phase dispersion range that can be achieved with the meta-atoms of metalens

Our proposed achromatic flat lens has the potential to expand applications in various fields. For instance, achromatic microlens arrays^41^ and metalens-arrays^16^ have shown significant benefits in light-field imaging. Our design can achieve higher performance broadband achromatic microlenses with higher NA and larger sizes, potentially enhancing resolution in light-field imaging. Imaging within the near-IR biological window is another significant application. Ref. ^30^ describes the design of nanopillars with a maximum phase dispersion of 1.97π and demonstrates a metalens with an NA of 0.1 and a diameter of 25 μm, capable of imaging within wavelengths from 650 nm to 1000 nm. Our approach can achieve higher equivalent maximum phase dispersion (13.60π), potentially allowing for higher resolution and large-aperture biological imaging. Additionally, a wider field of view can be achieved by optimizing the phase^34^ of our flat lens or expanding the aperture size as needed.

In conclusion, by zone division multiplexing of meta-atoms which is achieved by integrating meta-atoms onto a SPDC layer, we have successfully created a flat lens that boasts a high NA of 0.9 and a radius of 20.1 μm, demonstrating a broad achromatic bandwidth from 650 nm to 1000 nm in experiment. Compared to a conventional achromatic metalens with the same focal length and a maximum meta-atom phase dispersion of 2.20π, our achromatic flat lens enhances the maximum NA from 0.56 to 0.9 and increases the radius from 6.6 μm to 20.1 μm. In another aspect, conventional achromatic metalenses require an extremely high maximum phase dispersion of meta-atoms of at least 13.60π to deliver similar performance. Furthermore, we fabricated an achromatic flat lens with an NA of 0.7 and a radius of 30.0 μm, covering the same bandwidth, and achieved broadband microscopic imaging with an impressive resolution of 1.10 µm. This resolution surpasses that of both reported metalenses and MDLs, which do not achieve resolutions finer than 2 μm. In principle, our strategy can be at will enlarge the aperture size of the broadband achromatic flat lenses for any given focal length. This highlights the significant potential and versatility of lenses in advancing technological applications.

## Materials and methods

### Simulations

The meta-atoms within the metalens consist of c-Si nanopillars situated on a SiO_2_ substrate, arranged with a periodicity of 300 nm. To safeguard the structure, a layer of hydrogen silsesquioxane (HSQ) is applied and cured atop the nanopillars. The height of the c-Si nanostructures is established at 500 nm. The choice to use c-Si is driven by its high refractive index^51^, which allows the meta-atoms to demonstrate significant phase dispersion, and high transmission across the wavelength range from 650 nm to 1000 nm. To enhance phase dispersion further and ensure polarization insensitivity, five types of symmetrical meta-atoms are utilized, as shown in Fig. 2a. These meta-atoms achieve full 2π phase coverage and a maximum phase dispersion of 2.20π, as detailed in Fig. S1 of the Supplementary Information. Following this, the meta-atoms are organized according to the phase profile depicted in Fig. 1c and then are seated onto the SPDC layer according to the zone division. GaN is used as the material of the SPDC layer due to its low absorption and relatively high refractive index. This configuration yields to the achromatic flat lens illustrated in Fig. 1a, which boasts an NA of 0.9 and a radius of 20.1 μm.

FDTD simulations are performed using the commercial software FDTD Solutions (Lumerical Inc.). VDIM program is written in MATLAB.

### Fabrication

The fabrication process, detailed in Fig. S3 of the Supplementary Information, begins with patterning a set of gold (Au) markers on the gallium nitride (GaN) substrate using a lift-off technique for alignment purposes. A stepwise layer is then created at selected locations using focused ion beam (FIB) etching, guided by these markers. Next, a silica layer is grown on a Silicon-On-Insulator (SOI) substrate through chemical vapor deposition (CVD) and subsequently bonded to the GaN layer using bonding processing. The c-Si film on the SOI, with a thickness of 500 nm, is used in this process. Notably, during the bonding process, Norland Optical Adhesive 61 (NOA61) with a refractive index of 1.56 acts as the transparent medium between the SOI and GaN layers.

Following bonding, the silicon substrate of the SOI is removed by grinding and deep reactive ion etching (DRIE), while the oxide layer is eliminated using reactive ion etching (RIE) and hydrofluoric acid (HF) immersion, transferring the c-Si thin film onto the GaN layer. Another set of Au markers is then patterned on the c-Si surface, aligned with the initial Au markers on the GaN. The metalens pattern is aligned and defined on the c-Si film using negative resist hydrogen silsesquioxane (HSQ) under electron beam lithography (EBL), with the pattern subsequently transferred to the c-Si film via inductively coupled plasma (ICP) etching. Finally, HSQ is applied as a protective layer after spinning onto the film and undergoing a hard bake process at 200 °C.

### Optical characterization

The optical setup for focusing and imaging characterizations are illustrated in Fig. S4 of the Supplementary Information, respectively, employing a white light source (YSL, SC-pro 7) equipped with color filters to provide unpolarized incident light. For the focusing measurement, a polarizer is used to generate the x-polarized incident beam. A microscopic imaging system was configured using a high magnification objective lens (100×, Olympus), coupled with a tube lens, and a CMOS camera (Basler acA2040-90um) for assessing the performance of the device.

## Supplementary information

Supplementary Information for High-Performance Achromatic Flat Lens by Multiplexing Meta-Atoms on a Stepwise Phase Dispersion Compensation Layer

## Data Availability

All data and materials needed to evaluate the conclusions in the paper are present in the main text and the Supplementary Information. Additional data related to this paper may be requested from the corresponding authors.
