# Supplementary material for: High-performance achromatic flat lens by multiplexing meta-atoms on a stepwise phase dispersion compensation layer
Source: Light Sci Appl. 2025 Mar 5;14:110. doi: 10.1038/s41377-024-01731-8 (PMC11880545; doi:10.1038/s41377-024-01731-8)
Supplement: Supplementary file 1 — Supplementary Information for High-Performance Achromatic Flat Lens by Multiplexing Meta-Atoms on a Stepwise Phase Dispersion Compensation Layer [file 41377_2024_1731_MOESM1_ESM.docx]

Supplementary Information for

**High-Performance Achromatic Flat Lens by Multiplexing Meta-Atoms on a Stepwise Phase Dispersion Compensation Layer**

Jingen Lin ^1,4^, Jinbei Chen ^1,4^, Jianchao Zhang ^1,3^, Haowen Liang ^1,2*^, Juntao Li ^1,2*^,

Xue-Hua Wang ^1,2*^

^1^ State Key Laboratory of Optoelectronic Materials and Technologies, School of Physics, Sun Yat-Sen University, Guangzhou 510275, China.

^2^ Quantum Science Center of Guangdong-Hong Kong-Macao Greater Bay Area (Guangdong), Shenzhen, China.

^3^ Hisense Laser Display Co.,Ltd, 399 Songling Road, Qingdao, Shandong, China.

^4^ These authors contributed equally to this work.

*Corresponding authors. [lianghw26@mail.sysu.edu.cn](mailto:lianghw26@mail.sysu.edu.cn) (H.L.); [lijt3@mail.sysu.edu.cn](mailto:lijt3@mail.sysu.edu.cn) (J.Li); [wangxueh@mail.sysu.edu.cn](mailto:wangxueh@mail.sysu.edu.cn) (X.H.W.)

**Supplementary Discussion 1: The detailed design of the achromatic flat lens**

The required phase dispersions of the meta-atoms and the SPDC layer must satisfy Eq. (2) in the main text. For clarity, we rewrite it here:

(S1)

where is the required phase dispersion for the meta-atoms and its variation must be limited within . Here, Δ*L*(*r*) = *L*(*r*) - *L*(*R*) is the optical thickness difference of the SPDC layer between its radius at *r* and *R*. Obviously, the maximum phase dispersion occurs at *r*=0

$\Delta\Phi_{meta}+\frac{\Delta\omega}{c}\Delta L(0)=\frac{\Delta\omega}{c}\left[\sqrt{R^{2}+f^{2}}-f\right]$ (S2)

which results in the maximum radius *R* of the achromatic flat lens given by Eq. (3) in the main text

$\sqrt{R^{2}+f^{2}}=L_{meta}+f+\Delta L\left(0\right)$ (S3)

where *L_meta_* = *c*ΔΦ*_meta_* / Δ*ω* is the meta-featured size and $\Delta L\left(0\right)=\Delta nh_{1}$ (here *h*_1_ is the total height of the SPDC layer, $\Delta n$is the difference of the refractive index of two dielectrics in the SPDC layer).

It is demonstrated by Eq. (S3) that our strategy can freely enlarge the aperture sizes of the flat lenses by increasing the optical thickness difference$\Delta L\left(0\right)$of the SPDC layer between the center and the edge of the flat lens, without compromising the bandwidth Δ*ω* for a given ΔΦ*_meta_*. Of course, the experimental realization of the large $\Delta L\left(0\right)$ depends on the current fabrication technology.

When keeping $\Delta L\left(r\right)=\Delta L\left(0\right)$ unchanged, decreases with increasing *r* and down to zero at *r*_1_, i.e., the boundary of the center zone is determined by

$\sqrt{R^{2}+f^{2}}-\sqrt{r_{1}^{2}+f^{2}}-\Delta L\left(0\right)=0$ (S4)

Substituting Eq. (S3) into Eq. (S4), we have

$\sqrt{r_{1}^{2}+f^{2}}=L_{meta}+f$ (S5)

It is interesting to notice that $r_{1}$ is exactly the maximum radius of the conventional metalens without the SPDC layer (i.e., $\Delta L\left(r\right)=0$).

To enlarge the radius of the metalens, we introduce the SPDC layer for multiplexing the meta-atoms, where the optical thickness difference between adjacent zones decreases incrementally. We define the *i*-th zone of the SPDC layer as $r_{i-1}\leq r<r_{i}$ (*i* = 1, 2, …, N; $r_{0}=0$ and $r_{N}=R$) of the SPDC layer, the thickness of the *i*-th zone relative to *N*-th zone as $h_{i}$ ($h_{N}=0$). Then, the leaping variation of the optical thickness difference between the (*i-1*)-th and *i*-th zones can be expressed as

$\Delta L\left(r_{i}^{\text{-}}\right)-\Delta L\left(r_{\text{i+1}}^{\text{-}}\right)=\Delta L\left(r_{i}^{-}\right)-\Delta L\left(r_{i}^{+}\right)=\Delta n(h_{i}-h_{i+1}),\text{ }$ (S6a)

$\Delta L\left(r_{i}^{-}\right)=\Delta L\left(r_{i-1}^{+}\right)\text{, }(i=1,...,N-1)$ (S6b)

where $r_{i}^{_{\pm}}=r\pm\eta\text{ (}\eta\to0\text{)}$, $\Delta n$ is the difference between the high and the low refractive index in the SPDC layer. There are two different design schemes.

1. **Identical step-thickness scheme**

If we completely multiplex the library of the meta-atoms within each zone, i.e., the leaping variation $(\Delta\omega/c)\left[\Delta L\left(r_{i}^{-}\right)-\Delta L\left(r_{i}^{+}\right)\right]$ of the phase dispersion of the flat lens between adjacent zones and the variation of the phase dispersion within each zone are equal to$\Delta\Phi_{meta}$, we have from Eq. (S6a) and (S1)

$\Delta n(h_{i}-h_{i+1})=L_{meta}$ (S7)

$\sqrt{r_{i}^{2}+f^{2}}-\sqrt{r_{i-1}^{2}+f^{2}}=L_{meta}$ (S8)

Eqs. (S7) and (S8) show that the thickness of each step $\Delta h_{i}=h_{i}-h_{i+1}$ is identical, but its width $\Delta r_{i}=r_{i}-r_{i-1}$ is not. The total thickness$h_{1}$of the SPDC layer with *N* zones is

$\Delta nh_{1}=(N-1)L_{meta}$ (S9)

From Eq. (S5) and (S8), we have:

$\sqrt{r_{i}^{2}+f^{2}}=iL_{meta}+f$ (S10a)

$r_{i}^{2}=iL_{meta}(iL_{meta}+2f)$ (S10b)

By the iterating Eq. (S10b), we have:

$r_{i}^{2}-r_{i-1}^{2}=\left[\left(2i-1\right)L_{meta}+2f\right]L_{meta}$ (S11a)

$\Delta r_{i}=r_{i}-r_{i-1}=\frac{\left[\left(2i-1\right)L_{meta}+2f\right]L_{meta}}{r_{i}+r_{i-1}}$ (S11b)

When *i* is sufficiently large (*i →* ∞), we have *r_i_* ≈ *r_i-_*_1_ and Eq. (S11b) becomes:

$\Delta r_{i\to\infty}=\left.\frac{\left[\left(2i-1\right)L_{meta}+2f\right]L_{meta}}{2r_{i}}\right|_{i\to\infty}=\left.\frac{2iL_{meta}-L_{meta}+2f}{2iL_{meta}\sqrt{1+\frac{2f}{iL_{meta}}}}L_{meta}\right|_{i\to\infty}=L_{meta}$ (S12)

It is interesting to observe that the width$\Delta r_{i}$of each zone approaches the limit value of *L_meta_* if the aperture size is sufficiently large. This finding further demonstrates that the aperture sizes of flat lenses can be continually expanded, reinforcing the flexibility and scalability of our design strategy.

In our work, the SPDC layer is composed of silica (SiO_2_) and gallium nitride (GaN), with refractive indexes of 1.45 and 2.35, respectively. Substituting Δ*n* = 0.9 and$\Delta\Phi_{meta}\approx2.20\pi$ (Fig. S1) into the equation *L_meta_* = *c*ΔΦ*_meta_* / Δ*ω*, we obtain the meta-featured size of $L_{meta}=2.043$μm, the step thickness$\Delta h_{i}=2.27$μm and the total thickness $h_{1}=11.35$μm.

For the achromatic flat metalens with NA = 0.9 and *f* =9.7 μm, the required radius of *R*=20.0 μm is obtained by$NA=\sin[\arctan(R/f)]$. According to Eqs. (S10b), we get the radii as: *r*_1_= 6.6 μm, *r*_2_= 9.8 μm, *r*_3_= 12.5 μm, *r*_4_= 15.0 μm, *r*_5_= 17.4 μm, and *r*_6_=19.7 μm. It can be seen that *r*_6_=19.7 μm is the closest to the required radius *R*=20.0 μm. Therefore, six steps are sufficient for our design.

In experimental fabrication, the meta-atoms are typically arranged as periodic structures along the radial direction. However, using an identical step-thickness scheme results in varying step widths, which inevitably causes a mismatch between the edges of the side meta-atoms and the edges of the steps. This mismatch introduces unwanted diffraction, subsequently weakening the performance of the flat lenses. To avoid this problem, we adopt the following identical step-width scheme.

1. **Identical step-width scheme**

We divide the zone from *r*_1_ to the *R* required by NA and *f* with identical step-width into *N*-1 zones,

$r_{i}=r_{1}+(i-1)(R-r_{1})/(N-1)$ (S13)

Evidently, the radius $r_{i}$of the *i*-th zone is slightly different from that in the identical step-thickness scheme. It must be noted that Δ*r =* (*R* - *r*_1_) / (*N* - 1) should be the integer multiple *J* of the radial period *p* of the meta-atoms arranges to match the edges of the side meta-atoms with the edges of the steps, i.e. Δ*r = Jp*. From Eq. (S1), the required phase dispersion at each step edge *r_i_* is determined by

(S14)

In this scheme, we let the variation of the phase dispersion of the flat lens within each zone be equal to the leaping variation $(\Delta\omega\text{/c)}\Delta n\Delta h_{i}$ induced by the optical thickness difference between adjacent zones

(S15)

Therefore, the total height of the SPDC layer should be

$h_{1}=\left(\sqrt{R^{2}+f^{2}}-\sqrt{r_{1}^{2}+f^{2}}\right)/\Delta n$ (S16)

It is worth mentioning that in the identical step-width scheme, the required maximum phase dispersion of the meta-atoms in each zone slightly fluctuates around$\Delta\Phi_{meta}$.

Accordingly, this identical step-width scheme is utilized to accommodate the actual fabrication conditions in our laboratory. As the period *p* of a meta-atom is 300 nm (0.3 μm) in our design, we adopt *J* = 9, so that the identical step size equals to nine times the period *p*, i.e., Δ*r =* 2.7 μm, approximately equal to the step width of 2.68 μm determined by Eq. (S13). As a result, the fabricated radii of the steps from *r*_1_ to *r*_6_ are 6.6 μm, 9.3 μm, 12.0 μm, 14.7 μm, 17.4 μm, and 20.1 μm, where *r*_6_ = 20.1 μm is the closest to the required radius *R*=20.0 μm, as shown in Fig. S2. The corresponding thicknesses of steps are Δ*h_1_* = 1.89 μm, Δ*h_2_* = 2.22 μm, Δ*h_3_* = 2.42 μm, Δ*h_4_* = 2.57 μm, and Δ*h_5_* = 2.66 μm, resulting in a total thickness of the SPDC layer of 11.76 μm.

In the experiment, the accuracy of the focused ion beam (FIB) etching in our laboratory is approximately 0.1 μm, the actual thicknesses of steps were taken by Δ*h_1_* = 1.9 μm, Δ*h_2_* = 2.2 μm, Δ*h_3_* = 2.4 μm, Δ*h_4_* = 2.5 μm, and Δ*h_5_* = 2.6 μm. Therefore, the experimental total thickness of the SPDC layer is 11.6 μm.


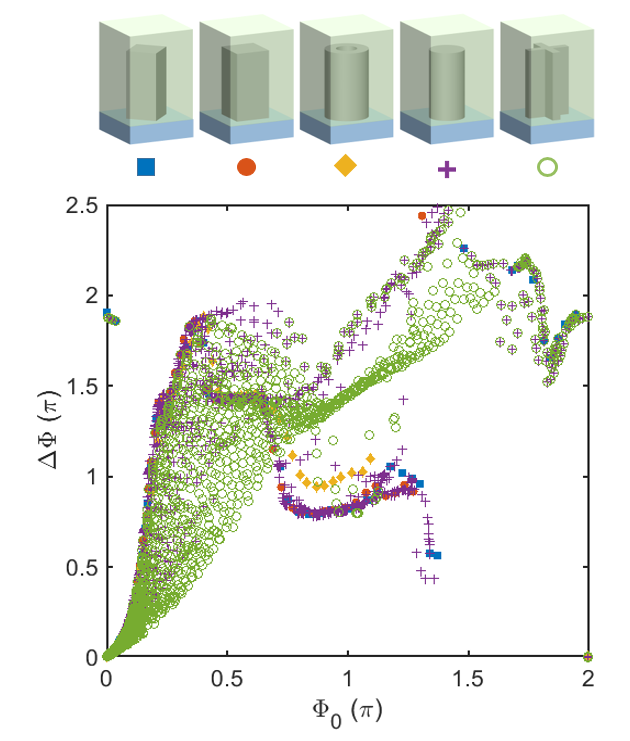


**Fig. S1.** The calculated phase Φ at a wavelength of 1000 nm and the corresponding phase dispersion $\Delta\Phi=\frac{d\Phi}{d\omega}\Delta\omega$ of the meta-atoms with a silicon nanopillar of 500 nm height. The bandwidth Δ*ω* corresponds to the achromatic wavelength range spanning from 650 nm to 1000 nm.


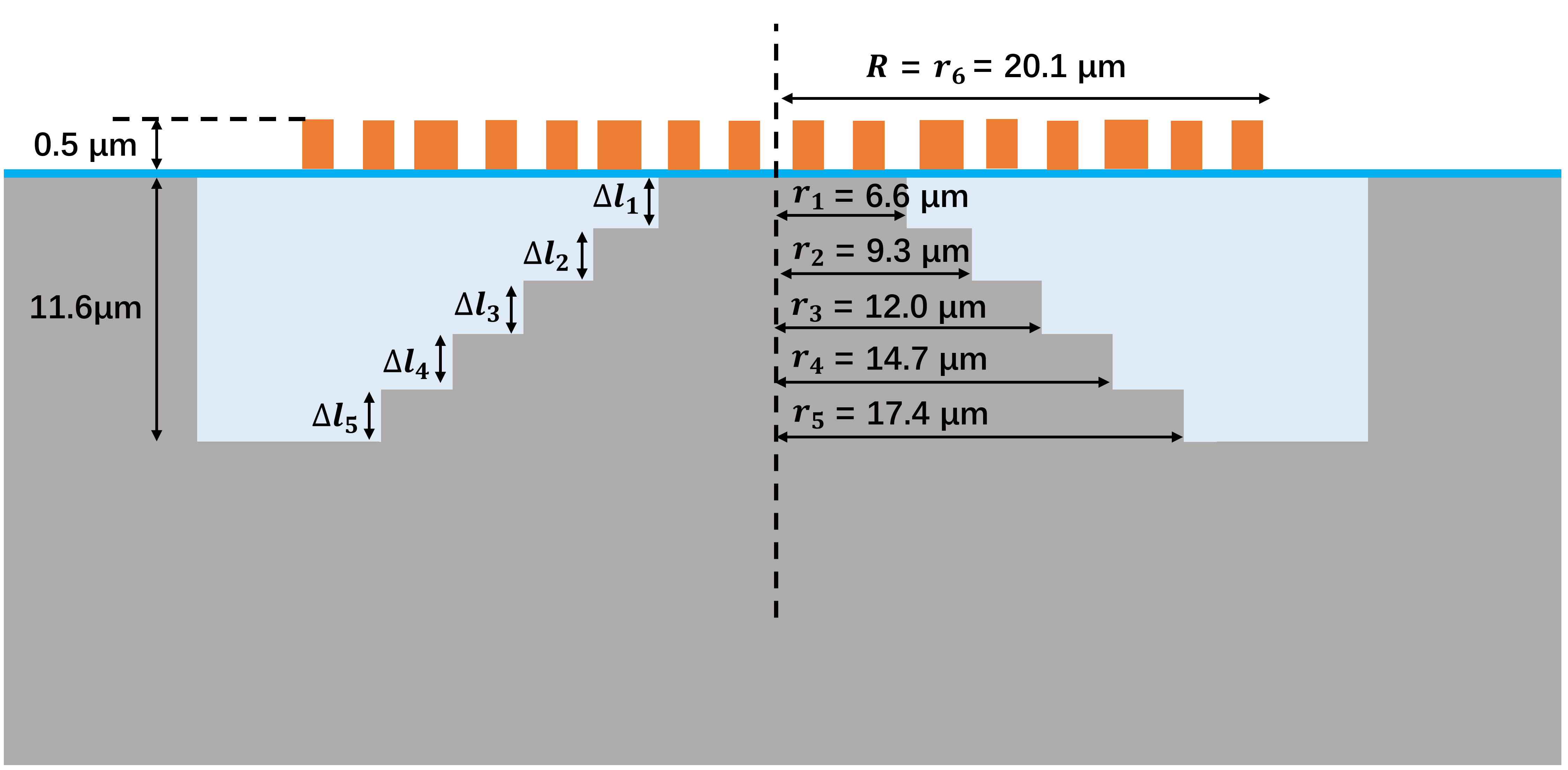


**Fig. S2.** Schematic of the designed achromatic flat lens with a NA of 0.9.

**Supplementary Discussion 2: The fabrication of the achromatic flat lens**


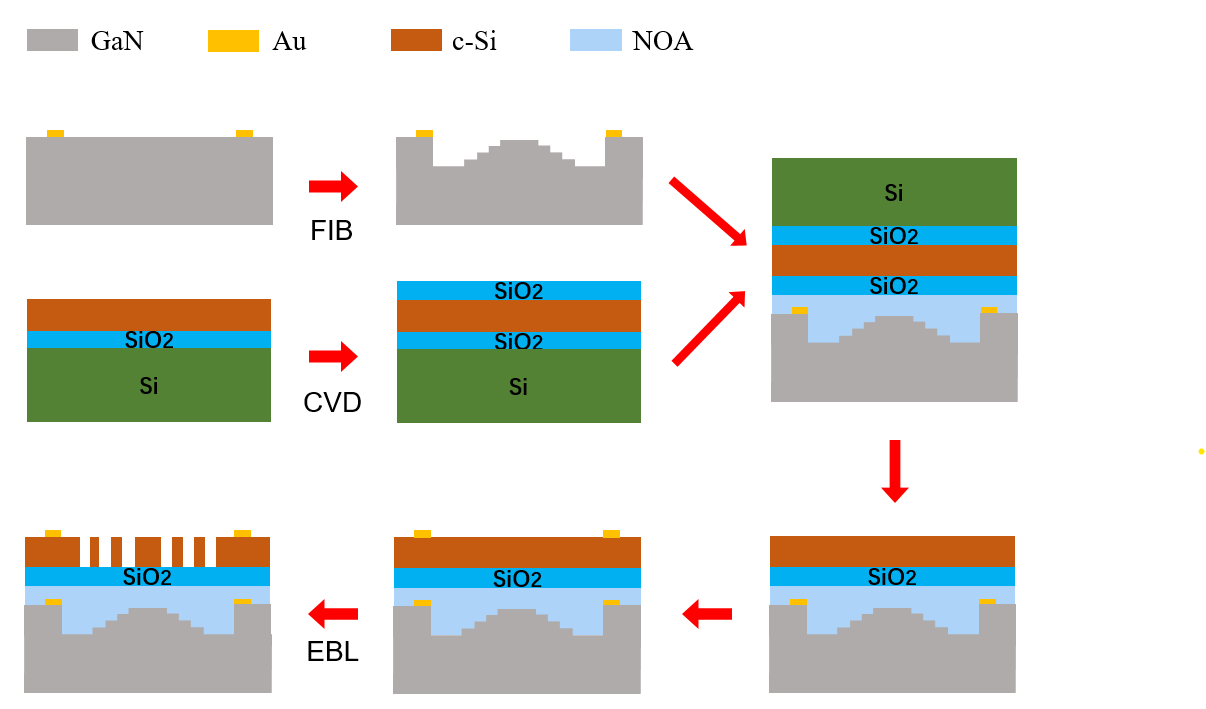


**Fig. S3.** Schematic illustration of the achromatic flat lens fabrication.

**Supplementary Discussion 3: The measurement of the achromatic flat lens**

To differentiate between the power transmitted through the lens and the power directed by the lens toward the focus, we define the focusing efficiency as the fraction of the incident light that passes through a circular aperture in the plane of focus with a radius equal to three times the FWHM spot size. For the achromatic flat lens with a NA of 0.9 and a radius of 20.1 μm, the simulated focusing efficiencies obtained via the FDTD method are 22.4%, 27.9%, 29.1%, 23.9%, and 25.9% at the wavelength of 650 nm, 700 nm, 800 nm, 900 nm and 1000 nm, respectively. It is noted that the efficiencies of flat lenses typically decrease as the NA increases. For very high NA values of 0.9, it is common for the efficiencies of achromatic flat lenses to be in the range of 20% to 30%, which is lower compared to monochromatic flat lenses. From this perspective, the efficiency of our proposed flat lens is consistent with expectations. The measured focusing efficiencies using the optical setup shown in Fig. S4a are 8.43%, 8.08%, 13.15%, 10.07% and 15.18% at the wavelength of 650 nm, 700 nm, 800 nm, 900 nm and 1000 nm, respectively. While for the achromatic flat lens with a NA of 0.7 and a radius of 30.0 μm, the measured focusing efficiencies are 7.1%, 7.8%, 9.2%, 11.3% and 10.4% at the same corresponding wavelengths. Fortunately, the experimental efficiency of about 10% is adequate to get high-resolution imaging, as shown in our work. The original captured images obtained by the optical setup depicted in Fig. S4b are presented in Fig. S5.


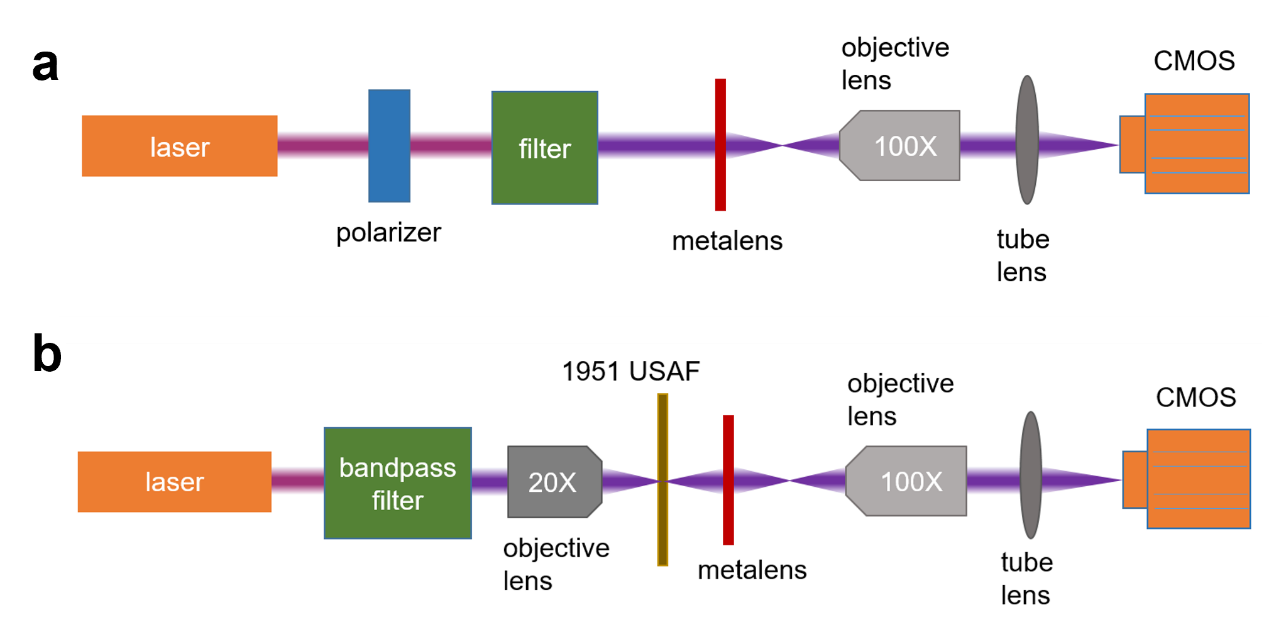


**Fig. S4.** Optical measurement setup for (**a**) focusing and (**b**) imaging.


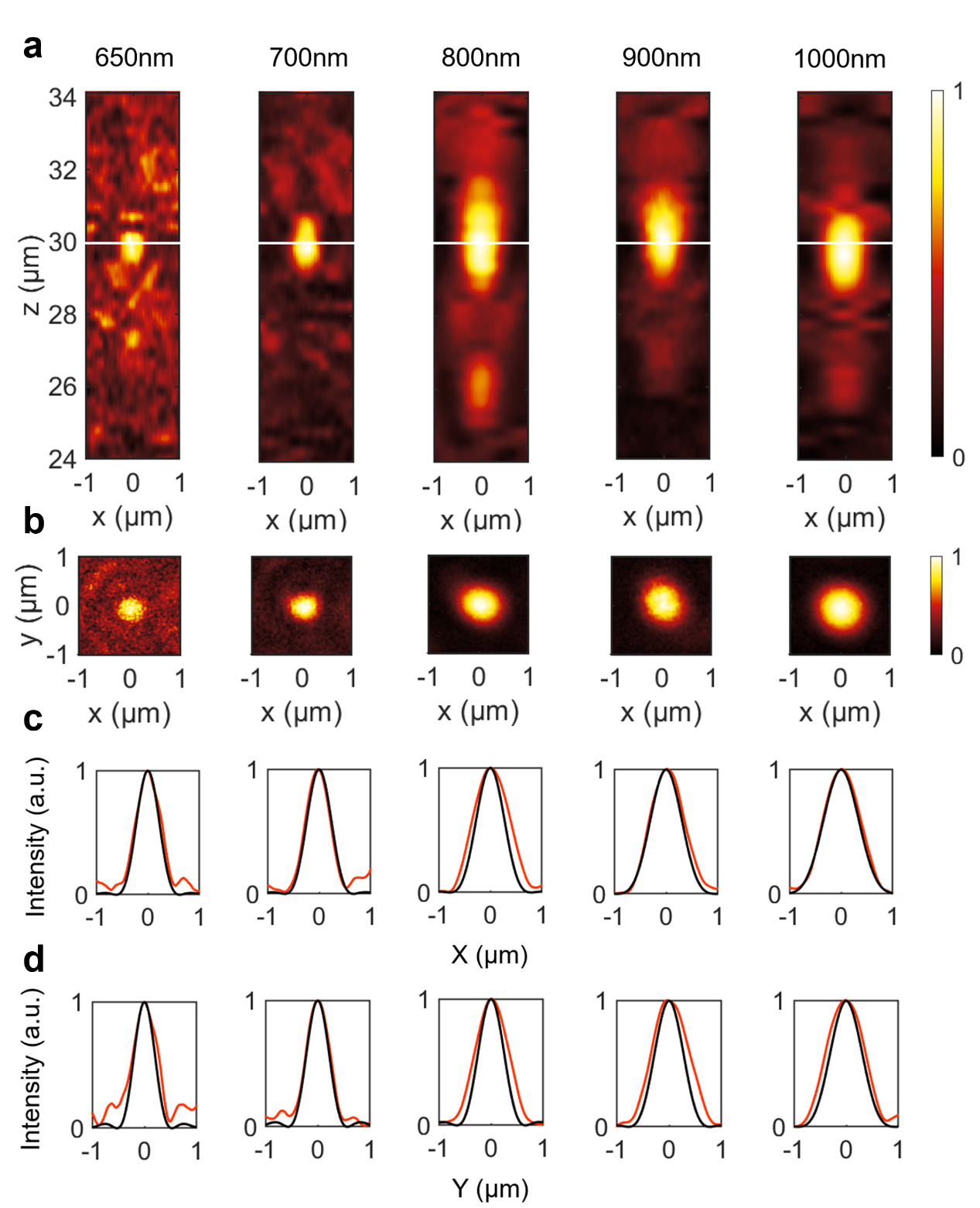


**Fig. S5. The measured PSFs of the achromatic flat lens with NA = 0.7 and a radius of 30.0 μm.** (**a**) Experimentally measured longitudinal PSFs at different incident wavelength by a x-polarized incident beam. The white lines indicate the focal plane. (**b**) The corresponding transverse PSFs at the focal plane. (**c**) The normalized intensity profiles at the focus plane in experimental (red lines) and in simulation through VDIM (black lines) at x direction. The FWHMs of the focal spot in experimental are respectively determined to be 575 nm, 571 nm, 820 nm, 839 nm, and 851 nm at wavelengths of 650 nm, 700 nm, 800 nm, 900 nm, and 1000 nm. (**d**) The normalized intensity profiles at the focus plane in experimental (red lines) and in simulation through VDIM (black lines) at y direction. The FWHMs of the focal spot in experimental are respectively determined to be 549 nm, 524 nm, 748 nm, 785 nm, and 848 nm at wavelengths of 650 nm, 700 nm, 800 nm, 900 nm, and 1000 nm.


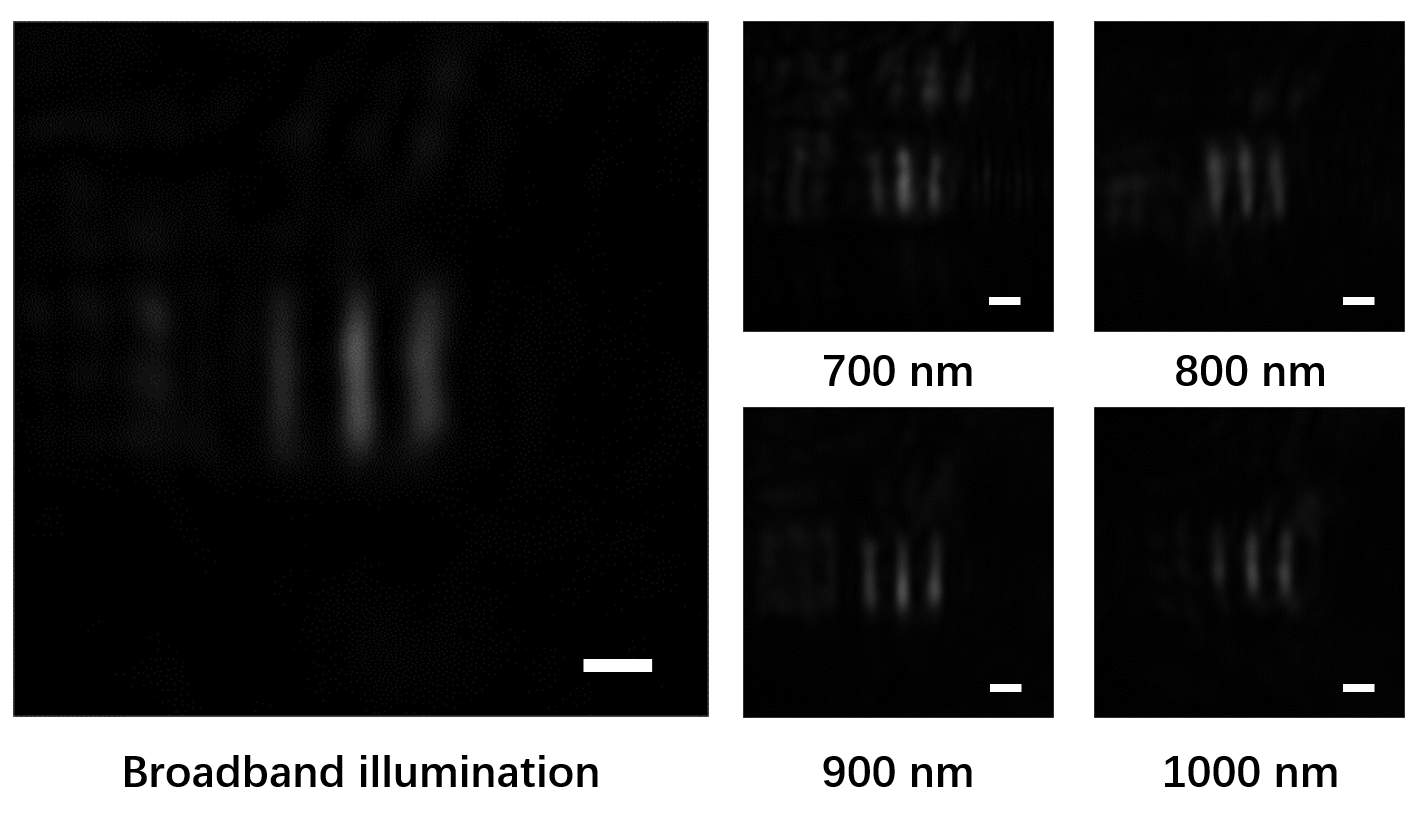


**Fig. S6.** The original image of element 6, group 8 of the 1951 Unites States Air Force resolution target under broadband, 700 nm, 800 nm, 900 nm and 1000 nm illumination, by using an achromatic flat lens with NA = 0.7 and a radius of 30.0 μm (Scale bar, 2 μm).

**Supplementary Discussion 4: Design of the SPDC layer for enlarging the aperture size of the achromatic flat lens at will**

To demonstrate the on-demand expansion of the aperture size for an achromatic flat lens with an achromatic bandwidth from 650 nm to 1000 nm according to the rules in Supplementary Discussion 1, we utilize the meta-atom library from our work and another one with much higher maximum phase dispersion. The meta-atom parameters of the two libraries are listed in Table S1.

**Table S1** The meta-atoms parameters of the two libraries

|  | Our meta-atoms | High dispersion meta-atoms |
| --- | --- | --- |
| ΔΦ*_meta_* | 2.20π | 7.00π |
| Periodicity (μm) | 0.30 | 0.65 |
| Height (μm) | 0.5 | 1.4 |
| Materials | GaN | c-Si |

Tables S2 and S3 list the geometric features of the achromatic flat lenses designed by the identical step-width scheme, with a numerical aperture (NA) of 0.9 and various radii constructed using the two libraries, respectively. According to the analysis in Supplementary Discussion 1, when the aperture size is sufficiently large, the step width$\Delta r_{i}$of each zone approaches the limit value of *L_meta_* if the maximum phase dispersion of the meta-atom library$\Delta\Phi_{meta}$is multiplexed. In this case, the *L_meta_* for the two libraries are 2.043 μm and 6.500 μm, respectively. On the other hand, the step width$\Delta r_{i}$ should be the integer multiple of the periodicity of the meta-atoms; therefore, the adopted step width should be 1.8 μm and 6.5 μm for the two cases, denoting 6 and 10 periods of meta-atoms contained within, respectively. Noting that the phase dispersion used in each zone would be slightly fluctuates around$\Delta\Phi_{meta}$. Following the design procedure of the identical step-width scheme, the *r*_1_ can be determined by Eq. (S8) with the known *f*. Then, the number of steps and the radius of the achromatic flat lens can be determined by Eq. (S13), and the total thickness of the SPDC layer is determined by Eq. (S16).

Tables S2 and S3 show that meta-atoms with larger maximum phase dispersion can effectively reduce the number of steps in the SPDC layer; however, the total thickness of the steps is roughly determined by the maximum phase dispersion, which is irrelevant to the type of meta-atom library used.

**Table S2** Geometric features of the achromatic flat lenses with NA of about 0.9 and various radii constructed by our meta-atoms

| Focal Length *f* (μm) | *r*_1_ (μm) | Total steps *N* | Identical step width *Δr* (μm) | Radius *R* (μm) | Total thickness of the steps *h*_1_ (μm) |
| --- | --- | --- | --- | --- | --- |
| 25 | 10.3 | 23 | 1.8 | 51.7 | 33.76 |
| 50 | 14.4 | 49 | 1.8 | 102.6 | 68.99 |
| 100 | 20.3 | 103 | 1.8 | 205.7 | 140.75 |

**Table S3** Geometric features of the achromatic flat lenses with NA of about 0.9 and various radii constructed by high dispersion meta-atoms

| Focal Length *f* (μm) | *r*_1_ (μm) | Total steps *N* | Identical step width *Δr* (μm) | Radius *R* (μm) | Total thickness of the steps *h*_1_ (μm) |
| --- | --- | --- | --- | --- | --- |
| 25 | 19.2 | 5 | 6.5 | 51.7 | 28.81 |
| 50 | 26.3 | 12 | 6.5 | 104.3 | 65.74 |
| 100 | 36.6 | 26 | 6.5 | 205.6 | 135.70 |

A much more significant aspect of our method is that it greatly reduces the thickness of large NA lenses compared to conventional objectives. To achieve broadband achromatism for an NA of 1.45, one typically needs to combine a meta-corrector and an objective, as described in Ref. [39]. This results in a total thickness determined by the size of the objective, and the lens is no longer a flat lens. In contrast, our method can achieve similar results on the scale of a flat lens. Table S4 lists the corresponding achromatic flat lenses constructed using our meta-atoms and high dispersion meta-atoms. The parameters of the objective correspond to a lens with an NA of 1.45 (in immersion oil) and an aperture radius of 393 μm. It shows that the total thickness of the SPDC layer is around 330 μm, which is much thinner than the size of a conventional objective. Increasing the refractive index contrast of the steps is able to achieve thinner total thickness.

**Table S4** Geometric features of the achromatic flat lenses with 1.45 NA constructed by our meta-atoms and high dispersion meta-atoms

|  | *Δn* | Focal Length *f* (μm) | *r*_1_ (μm) | Total steps *N* | Identical step width *Δr* (μm) | Radius *R* (μm) | Total thickness of the steps *h*_1_ (μm) |
| --- | --- | --- | --- | --- | --- | --- | --- |
| ours | 0.9 | 104 | 20.7 | 207 | 1.8 | 393.3 | 334.2 |
| ours | 2.3 | 104 | 20.7 | 207 | 1.8 | 393.3 | 130.8 |
| High dispersion meta-atoms of $\Delta\Phi_{meta}$=7.00π | 0.9 | 104 | 37.3 | 55 | 6.5 | 394.8 | 330.9 |
| High dispersion meta-atoms of $\Delta\Phi_{meta}$=7.00π | 2.3 | 104 | 37.3 | 55 | 6.5 | 394.8 | 129.5 |

Additionally, increasing the refractive index contrast of the steps, i.e. increasing the optical thickness of the SPDC layer, is also beneficial for reducing its total thickness. For instance, using crystalline silicon (c-Si, *n_c-Si_* ≈ 3.75 at 650 – 1000 nm) as a high index material results in an average refractive index contrast with the refilled SiO_2_ of approximately Δ*n* = 2.3. Table S4 also compares the parameters of the achromatic flat lenses formed by steps with different refractive index contrast for both our meta-atoms and the high dispersion meta-atoms, using the same focal length *f* = 104 μm. The results show that the total thickness of the SPDC layer can be significantly reduced by increasing Δ*n*.
